# Ultrasound-guided Platelet-rich Plasma Application Versus Corticosteroid Injections for the Treatment of Greater Trochanteric Pain Syndrome: A Prospective Controlled Randomized Comparative Clinical Study

**DOI:** 10.7759/cureus.6583

**Published:** 2020-01-07

**Authors:** Dimitrios Begkas, Stamatios-Theodoros Chatzopoulos, Panagiotis Touzopoulos, Alexia Balanika, Alexandros Pastroudis

**Affiliations:** 1 Orthopaedics, Asclepieion Voulas General Hospital, Athens, GRC; 2 Orthopaedics, University General Hospital of Alexandroupoli, Alexandroupoli, GRC; 3 Computed Tomography-Musculoskeletal Ultrasonography, Asclepeiion Voulas General Hospital, Athens, GRC

**Keywords:** platelet rich plasma, greater trochanteric pain syndrome

## Abstract

Purpose

The purpose of this clinical study was to evaluate and compare the effectiveness of ultrasound (US)-guided platelet-rich plasma (PRP) injections versus US-guided corticosteroid injections (CSI) in the treatment of greater trochanteric pain syndrome (GTPS).

Methods

Between January 2015 and December 2016, 24 patients with GTPS were enrolled and randomized in two groups (A and B). Group A (study group) patients received US-guided PRP injection treatment, while group B (control group) patients received US-guided CSI treatment. Clinical outcomes in both groups were evaluated and compared using the Visual Analogue Scale (VAS) of pain, the Harris Hip Score (HHS) and the presence or absence of complications at 4, 12, and 24 weeks post-injection. The level of significance was set at p<0.05.

Results

Both groups showed improved scores (VAS and HHS) compared to the pre-injection period, but patients in group A had a statistically significant (p <0.05) decrease in VAS score and a significantly increased HHS at the last follow-up (24 weeks post-injection). No complications were reported.

Conclusions

In conclusion, patients with GTPS present better and longer-lasting clinical results when treated with US-guided PRP injections compared to those with CSI. Further studies are needed to optimize the technical preparation of PRP, the sample concentration, the number of injections and the time intervals between them, in order to achieve the maximum desired results.

## Introduction

Greater trochanteric pain syndrome (GTPS), also known as trochanteric bursitis or gluteal tendinopathy, is a condition characterized by tenderness and pain over the greater trochanter (GT) radiating along the lateral border of the thigh [1]. It is more common in women aged 40 to 60 years, who are more frequently affected than men in the 4:1 ratio [2]. GTPS is the cause of pain in 10-20% of patients presenting to primary care with hip pain, with an incidence between 1.8 and 5.6 per 1000 per year [1-2].

GTPS can be caused by direct trauma, prolonged pressure on the hip, repetitive movements, unaccustomed vigorous exercise, long-lasting weight bearing on one leg, hip instability and sports injuries [3]. For many years, the reason for GTPS has remained unknown and several hypotheses have been proposed. Traditionally GTPS was thought to be caused by inflammation of the bursa (trochanteric bursitis), but surgical, histological, and imaging studies have shown that GTPS is attributable to tendinopathy of gluteal muscles (gluteus medius and/or gluteus minimus) and tensor fascia latae muscle. Inflammation of these tendons leads to secondary inflammation of the bursa [4]. Another cause is tightness of the iliotibial band, which also runs over the GT and can irritate the bursa associated with the gluteal tendons [4].

The main symptom of GTPS is lateral hip pain over the GT, which may radiate down the lateral thigh and the lower buttocks [3]. Pain may progressively worsen over time and be exacerbated when patient is lying on the affected side, with prolonged standing, sporting overuse, repetitive motions, weight-bearing, walking, long-distance running, falls and sitting with crossed legs [3-5].

The diagnosis of GTPS can be made firstly by physical examination, which is based on clinical tests. Single clinical tests lack validity, but a combination of them can be used to increase diagnostic accuracy. During an examination, two of them are most frequently used: the direct palpation ("jumping sign") and the 30-second single-leg stance test [6]. Other tests used are: resisted external rotation test, Trendelenburg test, FABER test, FADER test, Noble’s test, modified Ober’s test, Renne’s test, and ADD test [6-7].

GTPS must be differentially diagnosed from other common causes of hip pain like hip and knee osteoarthritis, lumbar spine pain, iliotibial band syndrome and other pelvic pathologies [8].

Radiological examination of the hip with x-rays is usually normal. Sometimes, it reveals calcification adjacent the GT and can also exclude common differentials including hip osteoarthritis, femoroacetabular impingement, and fractures [2]. Magnetic resonance imaging (MRI) and ultrasound (US) examination can detect peritrochanteric edema and gluteal tendinopathy. US can better detect calcifications than MRI and is very useful during aspiration and local injections [1]. MRI provides detailed soft tissue examination and can play an important role in differential diagnosis [1,7-8].

Management of GTPS remains unclear. The main goals are: to strengthen gluteal muscles, manage load and reduce compressive forces across GT, and treat comorbidities [2]. Most cases can be managed in primary care with rest, ice, taping, non-steroidal anti-inflammatory drugs (NSAIDs), weight loss, physical therapy, load modification and optimization of biomechanics [2]. Adjunct therapies include shock wave therapy and therapeutic ultrasound. Corticosteroid injections (CSI) can be effective in recalcitrant cases and give patients early pain relief [2,9]. Surgical intervention is used only if conservative treatment fails and involves bursectomy, tractus iliotibialis release, reduction osteotomy of the GT, and reconstruction of the abductor tendons [2,10-11].

Lately, platelet-rich plasma (PRP) has become very popular among the orthopaedic community as a minimally invasive method of enhancing tissue healing in conditions such as rotator cuff tears, patellar tendinopathy, lateral epicondylitis, osteochondral lesions, and knee osteoarthritis [11-15]. It has been proved that PRP promotes soft tissue healing by delivering a high concentration of platelet-derived growth factors to the diseased area and by enhancing cellular chemotaxis, proliferation and differentiation, removal of tissue debris, angiogenesis and lying down extracellular matrix [12,16].

Few papers in the literature refer to the treatment of GTPS with PRP. The purpose of this clinical study was to evaluate and compare the effectiveness of US-guided PRP injections versus CSI injections in the treatment of patients with GTPS.

## Materials and methods

This prospective, controlled, randomized, comparative clinical trial was approved by the Local Ethics Committee. All participating patients signed an informed consent form and were subjected to complete physical examination and imaging tests (x-rays, US, MRI), in order to confirm the diagnosis of GTPS and to rule out other pathologies.

The study was conducted in our clinic from January 2015 to December 2016 and included patients of both sexes over 18 years of age, who met the following criteria: tenderness and pain over the lateral hip and the greater trochanter for at least 12 weeks continuously, positive direct palpation and 30-second single-leg stance tests and no improvement with prior conservative treatment such as rest, ice, NSAIDs, shock wave and therapeutic US therapy etc. From the study were excluded patients who refused to participate in it, pregnant and lactating women and those who had recent hip injury, previous hip surgery, hip osteoarthritis, inflammatory disorders (rheumatoid arthritis, gout, ankylosing spondylitis etc.), lumbar spine problems, hematological and neurological disorders, cancer, diabetes, other pelvic pathologies, severe local or systematic infections, skin diseases, prior local therapy with corticosteroid injections, history of psychiatric illness and abuse of alcohol or drugs.

Among 62 patients with an initial diagnosis of GTPS, only 24 (18 women and 6 men) met the inclusion criteria. Their mean age was 48.7 (22-79) years and they were randomized into two clinical groups (A and B) of 12 patients of both sexes, according to their serial number (odd or even) assigned at the time of their first examination in the outpatient clinic. Group A patients (study group) were those with odd serial numbers, and they were treated with a single injection of 4 ml of PRP. Patients with even serial number were placed in group B (control group) and were treated with a single injection of 4 ml of methylprednisolone (40 mg/ml).

In all Group A cases, PRP preparation was performed in the operating theatre using the SW-PRP system provided by NTL Biologica. Under aseptic conditions, 40 ml of autologous blood was taken from the antecubital vein and added in a tube containing 6 ml of anticoagulant citrate dextrose solution. This was then placed into a SW-PRP device and centrifuged at 3850 rpm for seven minutes. Red blood cell fluid level in the device was optimized and then centrifuged for further four minutes at 3850 rpm. Platelet-poor plasma was extracted from the device and discarded. The remaining PRP was then extracted from the buffy coat layer and 4 ml of PRP were withdrawn and prepared for administration.

In order to increase the accuracy of targeting of the injections, all of them were guided by a US device with the aid of the same radiologist. The most painful place was identified by palpation. Patients were placed in the lateral decubitus position on their healthy side and with about 45 degrees of flexion of the affected hip. Just before the injection, the whole area was disinfected with povidon-iodine and alcohol solution and 3 ml of 2% lidocain hydrochloride (20mg/ml) were applied for local anesthesia of the skin and the subcutaneous tissue.

All patients were released 30 minutes after the injection. Broad-spectrum oral antibiotics and NSAIDs were prescribed for four days. They were allowed to immediately walk but were adviced to avoid strenuous activities for the first three days. Sports activities (jumping, running etc.), weight-bearing, and physical therapy were prohibited for at least four weeks.

Treatment outcomes of both groups were evaluated and compared using the Visual Analogue Scale (VAS), the Harris Hip Score (HHS), and the presence or absence of complications. Patients were evaluated before and at 4, 12, and 24 weeks post-injection.

Statistical analysis was done. The data were presented as mean and standard deviation. Calculations and statistics were performed with the Statistical Package of Social Science (SPSS 20) software. Statistical significance was set at p<0.05. 

## Results

Most of the 24 patients were middle-aged (41-50 years) (Table 1). There was a significant female dominance among them (75% women and 25% men, p <0.05) (Table 2). Post-injection (p.i.) follow-up was performed at 4-, 12-, and 24- week intervals and was completed by all patients. At the first follow-up (four weeks p.i.), both patient groups (A and B) showed improvement in pain scores, whereas group B showed better results than group A. However, at the second (12 weeks p.i.) and the third (24 weeks p.i.) follow-ups, group A showed significantly better pain scores compared to group B, which performed worse than at the first follow-up (Table 3). With regards the HHS, although both treatment groups at 4- and 12-weeks p.i. follow-ups showed much better results than at the pre-injection (pr.i.) period, at the third follow-up, the HHS in group A improved significantly as opposed to group B where HHS fell to pr.i. level (Table 4). Pain at the injection site was reported by 17 patients (70.8%) and lasted approximately three days. No further complications were reported.

**Table 1:** Age distribution of patients

Age (years)	Number of patients	Percent (%)	Group A (PRP)	Group B (Methylprednisolone)
21-30	1	4.2	1	0
31-40	5	20.8	2	3
41-50	9	37.5	5	4
51-60	5	20.8	2	3
>60	4	16.7	2	2
Total	24	100	12	12

**Table 2:** Distribution of patients according to their gender

Sex	Number of patients	Percent (%)	Group A (PRP)	Group B (Methylprednisolone)
Female	18	75	10	8
Male	6	25	2	4
Total	24	100	12	12

**Table 3:** Visual Analogue Scale (VAS) in group A and group B

VAS	Group A (PRP)		Group B (Methylprednisolone)			
	Mean	±SD	Mean	±SD	p value	Significance
VAS pre-injection	7.21	0.760	7.15	0.582	>0.05	Not significant
VAS 4 weeks p.i.	4.64	0.789	2.56	0.752	<0.05	Significant
VAS 12 weeks p.i.	3.16	0.866	6.57	0.915	<0.05	Significant
VAS 24 weeks p.i.	1.52	0.505	6.98	0.691	<0.05	Significant

**Table 4:** Harris Hip Score (HHS) in group A and group B

Group		Pre-injection	4 weeks p.i.	12 weeks p.i.	24 weeks p.i.
Group A (PRP)	Mean	55.91	80.83	86.12	96.17
	±SD	9.82	7.52	0.78	0.71
Group B (Methylprednisolone)	Mean	56.72	89.53	86.61	57.92
	±SD	9.93	6.02	4.71	11.13
	P value	>0.05	<0.05	<0.05	<0.05
	Significance	Not significant	Significant	Significant	Significant

## Discussion

GTPS is characterized by lateral hip pain and tenderness over the greater trochanter [1]. Most commonly involves middle-aged women [1-2]. Symptoms can be exacerbated by simple daily activities such as walking or lying on the side of the affected hip and can significantly affect and decrease patients' quality of life [3]. The underlying conditions in GTPS setting are tendinoses or tears of the gluteal tendons at the greater trochanter and not bursitis. Gluteus medius is most commonly affected than gluteus medius [4]. Thus, the treatment of GTPS should be directed to the treatment of the underlying tendon disorder.

In the vast majority of patients, treatment is effective with conservative measures such as rest, ice, NSAIDs and physical therapy. If conservative treatments fail, other more invasive options may be applied like local corticosteroid or PRP injections. Surgery is indicated only for recalcitrant cases, where all the other measures have been exhausted [2,9-11].

A traditional way of treating GTPS is percutaneous CSI. When applied superficially to tendinosis, in most cases, pain relief levels improve. Labrosse et al. reported 72% to 75% positive responses to pain at four weeks p.i. [17]. However, CSI have been shown to provide only short-term pain relief [2,9]. This can be explained by their analgesic effects on local neuropeptides and neurotransmitters that relay pain and not by their anti-inflammatory properties [16]. This is supported by histopathological studies that found that there is no association between gluteal tendinopathies and serious inflammations, which means that CSI cannot provide long-term pain relief [16,18]. Additionally, it was found that there is a risk of rupture if corticosteroids are injected into a tendon [12,19 ].

The use of PRP in treating tendinopathies has become popular in recent years. This is due to the fact that PRP contains concentrated platelets, biologically active molecules and proteins, which activate and accelerate the body's mechanisms of healing into forming new tissue [12]. PRP contains high concentrations of different growth factors like vascular endothelial growth factor (VEGF), insulin-like growth factor 1 (IGF-1), fibroblast growth factor (FGF), platelet-derived growth factor (PDGF) and transforming growth factor β1 (TGF-β1) [16]. VEGF stimulates angiogenesis of avascular chronically torn tendons [16]. IGF-1 stimulates collagen synthesis at the site of tendon injury [12,16]. FGF controls chemotaxis, cell proliferation and collagen synthesis. PDGF improves tendon matrix remodeling, stimulates collagen synthesis, and increases cell proliferation and chemotaxis [16]. These growth factors, in combination with anti-inflammatory components, activate the healing cascade and reverse the degenerative process [16].

Several in vivo and in vitro trials in human and animal models provided strong evidence of PRP promoting growth factor-mediated anabolic processes which are associated with proper tendon healing [13,20]. Also, multiple studies about using PRP in treating different tendinopathies have been reported [12-15]. Some of them tried to compare the outcomes of PRP administration with other kinds of treatment [12,21]. A small number of these studies talk about the application of PRP injections in the treatment of GTPS and only a few of them compare this type of treatment with the use of CSI [22-26]. The purpose of our study was to evaluate and compare the outcomes of US-guided PRP injections against CSI in the treatment of GTPS. This was achieved by applying a single US-guided injection (PRP or methylprednisolone) to each patient in the two groups and evaluating pain relief (VAS) and functional (HHS) outcomes at 4-,12- and 24-week follow-ups. Injection was the preferred method to apply PRP or methylprednisolone, and its US guidance ensured accurate localization and targeting of the tendon lesion.

In the present study, both groups of patients showed improvement in pain relief at the 4- and 12-week follow-ups. Group A patients were found to have significantly improved pain scores at 24-week follow-up compared with the CSI group (p<0.05). In group B, at the second follow-up (12 weeks p.i.) the VAS increased gradually as opposed to the first follow-up (four weeks p.i.) where it increased. At the end of 12 weeks p.i. the VAS score was not statistically different than that before the CSI application. Thus, we conclude that the pain relief effectiveness of CSI is only short-term. Our results agree with those of other studies where GTPS was treated with CSI, as well as with studies where CSI was used to treat other tendinopathies [12,25-26]. On the contrary, in group A, after 12 weeks, the VAS score remained significantly low and progressively decreased further at 24 weeks, which means that PRP efficacy is gradually improving and is significantly longer compared to CSI.

According to the HHS results, symptoms improved in both groups at the 4- and 12-week follow-ups. In group B, the HHS improved from 56.72 ± 9.93 to 89.53 ± 6.02 at four weeks p.i. and to 86.61 ± 4.71 at 12 weeks p.i., but dropped to near pr.i. level at 24 weeks p.i. (57.92±11.13). In contrast, the PRP group started with a pr.i. HHS of 55.91 ± 9.82 which increased to 80.83 ± 7.52 at four weeks p.i. and to 86.12 ± 0.78 at 12 weeks p.i. and presented further improvement at the final 24-week p.i. follow-up (96.17 ± 0.71). From the above results, we understand that in the treatment of GTPS, PRP injections are initially as effective as CSI, but unlike CSI, the efficacy of PRP does not deteriorate over time.

Our findings are in full agreement with those of previous studies [25-26]. However, Ribeiro et al., in a comparative study of PRP injections and CSI in the treatment of GTPS, found no significant differences in clinical outcomes [24]. This disagreement with our study could be explained by the fact that each study used different PRP-preparation equipment, different preparation methods, different spinning protocol and different platelet and growth factor concentrations. Another reason which could explain the difference in our results is the short-duration patient follow-up in the Ribeiro et al. study (only 60 days). As we have seen, in our study, PRP injections had slower onset action than CSI, but achieved much better pain relief and functional scores after the 12-week and 24-week follow-ups. That proved their long-term effectiveness. We assume that a longer follow-up period in the study of Ribeiro et al. could give different results closer to our findings.

In this study, no complications other than pain at the injection site (70.8% of patients) were reported, which means that both methods are safe if performed correctly, in the operating hall and under aseptic conditions. In addition, PRP has antimicrobial properties that help prevent infections [27]. However, there are studies that do not recommend the routine use of CSI due to their detrimental long-term effects [12,19]. Physical therapy was not allowed after the injection in both groups of patients, so any improvement found could be attributed to the action of the applied medicines.

There were some limitations in our study that need to be mentioned. The small sample size of patients and the short follow-up period limited the generalization of the final findings. The concentration of PRP in the prepared injection samples was not measured, depriving us of the opportunity to better understand the action of PRP. Our results were not confirmed by any imaging (MRI) or histopathological examination.

Although PRP is becoming an increasingly popular method of treating GTPS and other tendinopathies, the literature lacks studies with sufficient clinical data on functional outcomes, large sample sizes, long follow-up periods, and full explanation of the molecular and cellular mechanisms of action of PRP. There is also a need to establish formal protocols on the preparation and standardization of PRP and on the management of patients after treatment. All of the above indicate the necessity for further clinical and basic scientific research to meet these needs.

## Conclusions

In conclusion, patients with GTPS present better and longer-lasting clinical results when treated with US-guided PRP injections compared to those with CSI. Further studies are needed to optimize the technical preparation of PRP, the sample concentration, the number of injections and the time intervals between them, in order to achieve the maximum desired functional results.

## References

[1] Thomassen PJB, Basso T, Foss OA (2019). Endoscopic treatment of greater trochanteric pain syndrome - a case series of 11 patients. J Orthop Case Rep.

[2] Speers CJ, Bhogal GS (2017). Greater trochanteric pain syndrome: a review of diagnosis and management in general practice. Br J Gen Pract.

[3] Strauss EJ, Nho SJ, Kelly BT (2010). Greater trochanteric pain syndrome. Sports Med Arthrosc Rev.

[4] Grimaldi A, Mellor R, Hodges P, Bennell K, Wajswelner H, Vicenzino B (2015). Gluteal tendinopathy: a review of mechanisms, assessment and management. Sports Med.

[5] Fearon AM, Scarvell JM, Neeman T, Cook JL, Cormick W, Smith PN (2013). Greater trochanteric pain syndrome: defining the clinical syndrome. Br J Sports Med.

[6] Lequesne M, Mathieu P, Vuillemin-Bodaghi V, Bard H, Djian P (2008). Gluteal tendinopathy in refractory greater trochanter pain syndrome: diagnostic value of two clinical tests. Arthritis Rheum.

[7] Grimaldi A, Mellor R, Nicolson P, Hodges P, Bennell K, Vicenzino B (2017). Utility of clinical tests to diagnose MRI-confirmed gluteal tendinopathy in patients presenting with lateral hip pain. Br J Sports Med.

[8] Chowdhury R, Naaseri S, Lee J, Rajeswaran G (2014). Imaging and management of greater trochanteric pain syndrome. Postgrad Med J.

[9] Rothschild B (2013). Trochanteric area pain, the result of a quartet of bursal inflammation. World J Orthop.

[10] Govaert LH, van der Vis HM, Marti RK, Albers GH (2003). Trochanteric reduction osteotomy as a treatment of refractory trochanteric bursitis. J Bone Joint Surg Br.

[11] Davies JF, Stiehl JB, Davies JA, Geiger PB (2013). Surgical treatment of hip abductor tendon tears. J Bone Joint Surg Am.

[12] Upadhyay S, Damor V (2018). Autologous platelet rich plasma versus corticosteroid injection for chronic plantar fasciitis: a prospective controlled randomized comparative clinical study. Int J Res Med Sci.

[13] Schnabel LV, Mohammed HO, Miller BJ, McDermott WG, Jacobson MS, Santangelo KS, Fortier LA (2007). Platelet rich plasma (PRP) enhances anabolic gene expression patterns in flexor digitorum superficialis tendons. J Orthop Res.

[14] Di Matteo B, Filardo G, Kon E, Marcacci M (2015). Platelet-rich plasma: evidence for the treatment of patellar and Achilles tendinopathy: a systematic review. Musculoskelet Surg.

[15] Mautner K, Colberg RE, Malanga G (2013). Outcomes after ultrasound-guided platelet-rich plasma injections for chronic tendinopathy: a multicenter, retrospective review. PM R.

[16] Lee JJ, Harrison JR, Boachie-Adjei K, Vargas E, Moley PJ (2016). Platelet-rich plasma injections with needle tenotomy for gluteus medius tendinopathy: a registry study with prospective follow-up. Orthop J Sports Med.

[17] Labrosse JM, Cardinal E, Leduc BE (2010). Effectiveness of ultrasound-guided corticosteroid injection for the treatment of gluteus medius tendinopathy. AJR Am J Roentgenol.

[18] Silva F, Adams T, Feinstein J, Arroyo RA (2008). Trochanteric bursitis: refuting the myth of inflammation. J Clin Rheumatol.

[19] Chiavaras MM, Jacobson JA (2013). Ultrasound-guided tendon fenestration. Semin Musculoskelet Radiol.

[20] McCarrel T, Fortier L (2009). Temporal growth factor release from platelet-rich plasma, trehalose lyophilized platelets, and bone marrow aspirate and their effect on tendon and ligament gene expression. J Orthop Res.

[21] Arirachakaran A, Sukthuayat A, Sisayanarane T, Laoratanavoraphong S, Kanchanatawan W, Kongtharvonskul J (2016). Platelet-rich plasma versus autologous blood versus steroid injection in lateral epicondylitis: systematic review and network meta-analysis. J Orthop Traumatol.

[22] Ali M, Oderuth E, Atchia I, Malviya A (2018). The use of platelet-rich plasma in the treatment of greater trochanteric pain syndrome: a systematic literature review. J Hip Preserv Surg.

[23] Jacobson JA, Yablon CM, Henning PT (2016). Greater trochanteric pain syndrome: percutaneous tendon fenestration versus platelet-rich plasma injection for treatment of gluteal tendinosis. J Ultrasound Med.

[24] Ribeiro AG, Ricioli W Junior, Silva AR, Polesello GC, Guimaraes RP (2016). PRP in the treatment of trochanteric syndrome: a pilot study. Acta Ortop Bras.

[25] Fitzpatrick J, Bulsara MK, O'Donnell J, McCrory PR, Zheng MH (2018). The effectiveness of platelet-rich plasma injections in gluteal tendinopathy: a randomized, double-blind controlled trial comparing a single platelet-rich plasma injection with a single corticosteroid injection. Am J Sports Med.

[26] Jimenez-Pérez AE, Gonzalez-Arabio D, Diaz AS, Maderuelo JA, Ramos-Pascua LR (2019). Clinical and imaging effects of corticosteroids and platelet-rich plasma for the treatment of chronic plantar fasciitis: A comparative non randomized prospective study. Foot Ankle Surg.

[27] Badade PS, Mahale SA, Panjwani AA, Vaidya PD, Warang AD (2016). Antimicrobial effect of platelet-rich plasma and platelet-rich fibrin. Indian J Dent Res.

